# Impact of Early Versus Delayed Functional Endoscopic Sinus Surgery for Chronic Rhinosinusitis With Nasal Polyps

**DOI:** 10.1002/ohn.70131

**Published:** 2026-01-19

**Authors:** Radhika Duggal, Trisha Shang, David R. Hoying, David Kaelber, Mohamad R. Chaaban

**Affiliations:** ^1^ Cleveland Clinic Lerner College of Medicine of Case Western Reserve University Cleveland Ohio USA; ^2^ Department of Otolaryngology–Head and Neck Surgery Cleveland Clinic Head and Neck Institute Cleveland Ohio USA; ^3^ Case Western Reserve University School of Medicine Case Western Reserve University Cleveland Ohio USA; ^4^ Departments of Internal Medicine, Pediatrics, and Population and Quantitative Health Sciences Case Western Reserve University Cleveland Ohio USA

**Keywords:** biologic therapy, chronic rhinosinusitis, chronic rhinosinusitis with nasal polyps, disease control, dupilumab, functional endoscopic sinus surgery, mepolizumab, nasal polyps, omalizumab, surgical timing

## Abstract

**Objective:**

Previous studies have suggested a benefit to early functional endoscopic sinus surgery (FESS) for chronic rhinosinusitis (CRS). With the approval of T2 biologics for CRS with nasal polyps (CRSwNP), it is important to re‐evaluate FESS timing in relation to T2 biologic utilization. In this study, we aimed to understand the impact of FESS timing on CRSwNP disease control.

**Study Design:**

Retrospective database study.

**Setting:**

Patients diagnosed with CRSwNP in the TriNetX platform.

**Methods:**

The TriNetX platform was queried to identify adult patients with one or more encounter diagnoses of CRSwNP. Among this cohort, we identified subgroups of individuals who underwent early (within 1 year) or delayed (1‐2, 2‐3, 3‐4, 4‐5, or >5 years after initial encounter) FESS, based on CPT codes. Pertinent outcomes, including the need for revision FESS and first‐time T2 biologic prescription, were compared.

**Results:**

After 1:1 propensity matching, we included a range of 264 to 1419 patients in each cohort. When followed for 2 and 5 years postoperatively, the delayed FESS (>5 years) cohort had 96% and 83% greater odds of new T2 biologic prescription (OR 1.96, 95% CI 1.40‐2.73 and OR 1.83, 95% CI 1.40‐2.40), but had no significant difference in the odds of revision FESS (2 year OR 1.03, 95% CI 0.73‐1.47 and 5 year OR 1.23, 95% CI 0.93‐1.63).

**Conclusions:**

Our findings demonstrate that the delayed FESS cohort had a higher T2 biologic prescription rate. Additional investigations are necessary to determine if this is due to the recent introduction of T2 biologics or a consequence of heightened disease severity necessitating long‐term biologic use when surgery is delayed.

Chronic rhinosinusitis (CRS) is a chronic inflammatory disease of the paranasal sinuses lasting at least 12 weeks with symptoms including nasal obstruction, nasal congestion, mucopurulent nasal discharge, facial pain/pressure, and hyposmia.^1^ Among all patients with CRS, 25% to 30% concurrently have nasal polyps, classified as chronic rhinosinusitis with nasal polyps (CRSwNP).^2^ Management for CRSwNP not controlled with appropriate medical therapy measures has evolved in recent years, now including both medical management with FDA‐approved biologics, such as dupilumab, mepolizumab, and omalizumab, and functional endoscopic sinus surgery (FESS).^3^, ^4^, ^5^

Previous studies have demonstrated that a prolonged interval between diagnosis and FESS for CRS may negatively impact outcomes, including a reduction in improvement of SNOT‐22 score,^6^ increase in incidence of new asthma diagnosis,^7^ and increase in healthcare utilization.^8^, ^9^ Furthermore, the longevity of surgical benefit in patients with delayed FESS has been found to be shorter than that found in patients who received early FESS.^6^ Prior studies have suggested that early intervention contributes to improved outcomes by reducing the time during which sinonasal tissues undergo remodeling secondary to the underlying the inflammatory pathophysiology of CRS.^10^, ^11^

While studies have established a relationship between early FESS and improved outcomes, to our knowledge, no study has examined the impact of FESS timing on the need for T2 biologics in addition to revision FESS in the setting of CRSwNP. Given the long‐term revision rate of FESS among patients with CRSwNP is estimated as high as 40%,^12^, ^13^ and that multiple patient factors have previously been associated with a greater likelihood of need for revision FESS,^14^ it is important to further characterize the role of FESS timing. This information may inform future recommendations regarding optimal timing of FESS for patients with CRSwNP. Therefore, in this study, we utilized the TriNetX Analytics platform to understand the relationship between timing of FESS and need for additional disease management, including revision FESS and T2 biologic utilization in patients with CRSwNP.

## Methods

The US Collaborative Network within the TriNetX Analytics platform consists of deidentified electronic health record (EHR) data of more than 100 million patients in 65 healthcare organizations. This data platform has been deemed exempt from the Western Institutional Review Board (IRB) by a qualified expert as defined in Section §164.514(b)(1) of the HIPAA Privacy Rule. Our study protocol for the use of the TriNetX platform was also approved as exempt human subject research by the Cleveland Clinic IRB. This study utilized the International Statistical Classification of Diseases and Related Health Problems, Tenth Revision (ICD‐10), along with Logical Observation Identifier Names and Codes (LOINC), Department of Veteran Affairs Drug Classification (VA) Codes, Anatomical Therapeutic Chemical Codes, RxNorm Codes, and electronic health record (EHR) demographics, to define our cohorts, matched variables, and outcomes. The specific codes utilized to define the cohorts and outcomes are reported within the Supporting Materials.

### Patient Cohorts

The TriNetX platform was queried on July 14, 2025 for all patients within the database with a first instance ICD‐10 diagnosis of CRS (ICD‐10 J32) with nasal polyps (ICD‐10 J33) who underwent their initial FESS (CPT 31254‐31288). Patients were then grouped by time interval from initial diagnosis to primary FESS. There was a total of 6 groups including intervals <1 year, 1 to 2 years, 2 to 3 years, 3 to 4 years, 4 to 5 years and >5 years. Patients in each cohort were matched by sex and age.

### Outcome Variables

The purpose of this retrospective analysis was to assess the impact of performing FESS < 1 year, 1 to 2 years, 2 to 3 years, 3 to 4 years, 4 to 5 years, and >5 years after initial ICD‐10 diagnosis of CRSwNP on revision FESS rates (CPT 31254‐31288), first‐time T2 biologic prescriptions (dupilumab, mepolizumab, and omalizumab), combined rates of T2 biologic prescriptions and revision FESS, number of instances of post‐operative healthcare utilization related to sinus disease, and number of instances of sinusitis/nasal polyps related prescriptions. The postoperative healthcare utilization outcome metric consisted of all visits with ICD‐10 diagnosis codes of sinusitis (ICD‐10 J01 or J32) and nasal polyps (ICD‐10 J33) as well as visits with procedure codes related to sinus disease (CPT 30000 to 31999). Postoperative prescriptions related to sinusitis/nasal polyps consisted of the following medication classes: antibiotics, antifungals, antihistamines, antitussives, antivirals, bronchodilators, corticosteroids, leukotriene modifiers, pain management, and nasal steroids. Each time period was compared to the early FESS cohort (<1 year) to assess the difference in outcomes within the first year after initial FESS.

### Statistical Analysis

All statistical analyses were conducted using TriNetX′s built‐in advanced analytics tools for cohort comparisons and totaling of outcomes. Odds ratios (OR) between the compared time periods were calculated with a 95% confidence interval (CI). The TriNetX platform performed 1:1 propensity score matching based on age and sex between the compared cohorts through logistic regression, employing nearest‐neighbor matching. A matching tolerance level of 0.01 and a standardized mean difference (SMD) of 0.1 or less between covariates were used. Baseline and matched patient characteristics were compared using chi‐squared tests for categorical variables and independent sample *t*‐tests for continuous variables. All analyses and queries for post‐FESS outcomes were conducted on July 14, 2025.

## Results

Overall, 24,460 patients were identified. Baseline demographics prior to 1:1 propensity matching are depicted in Table 1, with bolded values indicating characteristics which differ in comparison to the early FESS cohort. After 1:1 propensity matching based on age and sex, our analysis included a range of 264 to 1419 patients in each cohort, dependent on which cohorts were matched. Supplemental Tables S1‐5, available online further enumerate baseline comorbidities across all delayed FESS groups when compared to normal, and demonstrate the standardized mean difference (SMD) across each factor before and after propensity matching.

**Table 1:** Baseline Demographics Before Propensity Matching at Time of FESS (<1 yr, 1‐2 yr, 2‐3 yr, 3‐4 yr, 4‐5 yr, >5yr)

Characteristic	<1 year (n = 20,382)	1‐2 years (n = 1,223)	2‐3 years (n = 602)	3‐4 years (n = 423)	4‐5 years (n = 292)	>5 years (n = 1538)
Age at Index (mean ± SD)	47 ± 17.5	48 ± 17	48.5 ± 16.9	49.4 ± 17.1	51.0 ± 16.6	47.0 ± 17.5
Male (%)	59.8%	58.1%	55.9%	59.5%	57.6%	56.2%
Allergic rhinitis, unspecified (%)	27.9%	**39.6%**	**45.3%**	**46.1%**	**48.8%**	**57.6%**
Asthma (%)	33.7%	**45.3%**	**33.7%**	**52.0%**	**52.7%**	**60.2%**
Depressive episode (%)	9.9%	12.0%	14.5%	14.4%	**16.3%**	**21.9%**
Diabetes mellitus (%)	10.7%	11.9%	**14.3%**	13.7%	14.8%	**17.5%**
Type 2 Diabetes mellitus	10.4%	11.3%	**13.6%**	13.4%	14.1%	**16.1%**
Sleep apnea	14.1%	**15.9%**	**18.3%**	18.3%	19.4%	**23.3%**
Chronic ischemic heart disease	6.1%	7.2%	8.4%	6.6%	9.5%	**10.8%**
Essential (primary) hypertension	29.0%	32.1%	35.5%	38.3%	38.9%	**41.8%**
Diseases of the circulatory system	38.2%	42.6%	46.0%	46.8%	46.6%	**60.2%**
Normal weight (BMI < 30)	31.2%	31.5%	33.3%	**37.6%**	**41.7%**	**44.3%**
Sinus surgery extent (CPT)						
Maxillary antrostomy w/tissue removal (31,267)	68.6%	64.4%	64.8%	62.2%	59.7%	64.9%
Frontal sinus w/tissue removal (31,276)	51.3%	52.5%	52.3%	49.5%	53.0%	50.2%
Total ethmoidectomy (31,255)	39.2%	39.0%	39.9%	43.2%	47.0%	32.0%
Maxillary antrostomy (31,256)	28.0%	31.2%	27.4%	26.8%	30.4%	30.0%
Total ethmoidectomy and sphenoidotomy w/removal of tissue (31,259)	25.7%	27.3%	27.0%	24.4%	20.8%	27.0%
Sphenoidotomy w/removal of tissue (31,288)	16.5%	13.7%	17.4%	16.1%	15.2%	13.7%
Sphenoidotomy (31,287)	15.7%	17.1%	15.3%	18.0%	21.2%	15.6%
Total ethmoidectomy with frontal sinus exploration w/removal of tissue (31,253)	15.3%	15.2%	14.1%	16.8%	14.8%	18.1%
Partial Ethmoidectomy (31,254)	13.9%	11.3%	11.8%	9.3%	12.4%	11.1%
Total Ethmoidectomy and Sphenoidotomy (31,257)	11.2%	11.8%	14.5%	12.0%	11.7%	14.3%

The TriNetX U.S. Collaborative Network is a live database, and values are captured from one point in time. Bolded values indicate statistical significance (*P* < .05) when compared to <1 year cohort.

We compared outcomes of revision FESS and new T2 biologic prescription within 2 years of primary FESS. Tables 2 and 3 demonstrate the odds of revision FESS and new T2 biologic prescription, respectively, within 2 years of primary FESS. In our analysis of both outcomes, a significant difference was only noted in the delayed FESS (>5 years) group relative to the early FESS group. We found that individuals undergoing delayed FESS (>5 years) had a 96% increase in the odds of T2 biologic prescription (OR 1.96, 95% CI: 1.40, 2.73). Table 4 demonstrates the odds of the combined outcomes of revision FESS and new T2 biologic prescription within 2 years of primary FESS. We found that patients undergoing delayed FESS had a greater odds of this cumulative outcome, with individuals undergoing delayed FESS (>5 years) having a 83% increase in the odds of revision FESS or new T2 biologic prescription (OR 1.83, 95% CI 1.44‐2.31).

**Table 2:** Delayed FESS Verus Early FESS: Revision FESS Within 2 Years Postoperatively

Delayed FESS (number of years)	% (n/N) patients with outcome in delayed FESS group	% (n/N) patients with outcome in matched early FESS group (<1 yr after initial CRSwNP diagnosis)	Odds ratio (95% CI)	*P* value
1‐2 years	5.0% (59/1173)	4.8% (56/1173)	1.06 (0.73, 1.54)	.77
2‐3 years	5.2% (30/574)	4.4% (25/574)	1.21 (0.70, 2.09)	.49
3‐4 years	4.1% (17/410)	3.7% (15/410)	1.14 (0.56, 2.31)	.72
4‐5 years	4.6% (13/283)	3.9% (11/283)	1.19 (0.52, 2.70)	.68
>5 years	4.6% (65/1419)	4.4% (63/1419)	1.03 (0.73, 1.47)	.86

N size has been 1:1 propensity‐matched for each cohort comparison.

**Table 3:** Delayed FESS Verus Early FESS: New Biologic Prescription Within 2 Years Postoperatively

Delayed FESS (number of years)	n/N patients with outcome in delayed FESS group	n/N patients with outcome in matched early FESS group (<1 yr after initial CRSwNP diagnosis)	Odds ratio (95% CI)	*P* value
1‐2 years	5.5% (61/1110)	4.0% (46/1158)	1.41 (0.95, 2.08)	.09
2‐3 years	5.8% (32/550)	5.3% (30/570)	1.11 (0.67, 1.86)	.69
3‐4 years	3.6% (14/387)	4.9% (20/407)	0.73 (0.36, 1.46)	.37
4‐5 years	4.2% (11/264)	3.5% (10/282)	1.18 (0.49, 2.83)	.71
>5 years	7.8% (102/1314)	4.1% (58/1406)	1.96 (1.40, 2.73)	<.001*

N size has been 1:1 propensity‐matched for each cohort comparison. *n/a = 1‐10, so results are too few to run analysis. Bolded value indicates statistical significance (*P* < .05).

**Table 4:** Delayed FESS Verus Early FESS: Combined Revision FESS or New Biologic Prescription Within 2 Years Postoperatively

Delayed FESS (number of years)	n/N patients with outcome in delayed FESS group	n/N patients with outcome in matched early FESS group (<1 yr after initial CRSwNP diagnosis)	Odds ratio (95% CI)	*P* value
1‐2 years	12.0% (141/1173)	9.0% (105/1173)	1.39 (1.06, 1.82)	.02*
2‐3 years	12.7% (73/574)	9.4% (54/574)	1.40 (0.97, 2.04)	.07
3‐4 years	10.5% (43/410)	8.5% (35/410)	1.26 (0.79, 2.01)	.34
4‐5 years	12.4% (35/283)	7.4% (21/283)	1.76 (1.00, 3.11)	.049*
>5 years	14.7% (208/1419)	8.6% (122/1419)	1.83 (1.44, 2.31)	<.001*

N size has been 1:1 propensity‐matched for each cohort comparison. Bolded values indicate statistical significance (*P* < .05).

We further compared outcomes of revision FESS and new T2 biologic prescription within 5 years of primary FESS. Table 5 demonstrates the comparisons of revision FESS, and showed only a statistically significant difference between delayed (1‐2 year) FESS and early FESS groups (OR 1.49, 95% CI 1.11‐2.00). Table 6 demonstrates the odds of new T2 biologic prescription within 5 years of primary FESS. There was a significant difference in the 1 to 2 years delayed FESS group (OR: 1.39, 95% CI: 1.02, 1.90) and >5 years (OR: 1.83, 95% CI: 1.40, 2.40) each compared to early FESS. Finally, Table 7 shows the odds of the combined outcomes of revision FESS and new T2 biologic prescription within 5 years of primary FESS. There was a significant difference in all delayed FESS groups when compared to early FESS for the combined outcome. The 1 to 2 years delayed group had an OR of 1.58 (95% CI: 1.26‐1.98), the 2 to 3 years delayed group had an OR of 1.54 (95% CI: 1.12‐2.11), the 3 to 4 years delayed group had an OR of 1.56 (95% CI: 1.06‐2.30), the 4 to 5 years delayed group had an OR of 1.94 (95% CI 1.21‐3.10) and the >5 years delayed group had an OR of 1.84 (95% CI: 1.50‐2.25) each compared to the early FESS group.

**Table 5:** Delayed FESS Versus Early FESS: Revision FESS Within 5 Years Postoperatively

Delayed FESS (number of years)	n/N patients with outcome in delayed FESS group	n/N patients with outcome in matched early FESS group (<1 yr after initial CRSwNP diagnosis)	Odds ratio (95% CI)	*P* value
1‐2 years	10.1% (118/1173)	7.0% (82/1173)	1.49 (1.11, 2.00)	.008*
2‐3 years	9.9% (57/574)	7.0% (40/574)	1.47 (0.97, 2.25)	.07
3‐4 years	9.0% (37/410)	5.9% (24/410)	1.60 (0.94, 2.72)	.08
4‐5 years	8.8% (25/283)	5.7% (16/283)	1.62 (0.84, 3.10)	.14
>5 years	8.2% (116/1419)	6.8% (96/1419)	1.23 (0.93, 1.63)	.15

N size has been 1:1 propensity‐matched for each cohort comparison. Bolded value indicates statistical significance (*P* < .05).

**Table 6:** Delayed FESS Versus Early FESS: New Biologic Prescription Within 5 Years Postoperatively

Delayed FESS (number of years)	n/N patients with outcome in delayed FESS group	n/N patients with outcome in matched early FESS group (<1 yr after initial CRSwNP diagnosis)	Odds ratio (95% CI)	*P* value
1‐2 years	8.9% (99/1110)	6.6% (76/1158)	1.39 (1.02, 1.90)	.04*
2‐3 years	8.7% (48/550)	7.7% (44/570)	1.14 (0.75, 1.75)	.54
3‐4 years	6.5% (25/387)	6.9% (28/407)	0.94 (0.54, 1.63)	.81
4‐5 years	8.0% (21/264)	5.7% (16/282)	1.44 (0.73, 2.82)	.29
>5 years	11.5% (151/1314)	6.6% (93/1406)	1.83 (1.40, 2.40)	<.001*

N size has been 1:1 propensity‐matched for each cohort comparison. Bolded values indicate statistical significance (*P* < .05).

**Table 7:** Delayed FESS Versus Early FESS: Combined Revision FESS or New Biologic Prescription Within 5 Years Postoperatively

Delayed FESS (number of years)	n/N patients with outcome in delayed FESS group	n/N patients with outcome in matched early FESS group (<1 yr after initial CRSwNP diagnosis)	Odds ratio (95% CI)	*P* value
1‐2 years	18.8% (221/1173)	12.8% (150/1173)	1.58 (1.26, 1.98)	<.001*
2‐3 years	19.5% (112/574)	13.6% (78/574)	1.54 (1.12, 2.11)	.007*
3‐4 years	17.8% (73/410)	12.2% (50/410)	1.56 (1.06, 2.30)	.02*
4‐5 years	19.8% (56/283)	11.3% (32/283)	1.94 (1.21, 3.10)	.005*
>5 years	20.9% (296/1419)	12.5% (178/1419)	1.84 (1.50, 2.25)	<.001*

N size has been 1:1 propensity‐matched for each cohort comparison. Bolded values indicate statistical significance (*P* < .05).

**Figure 1:**
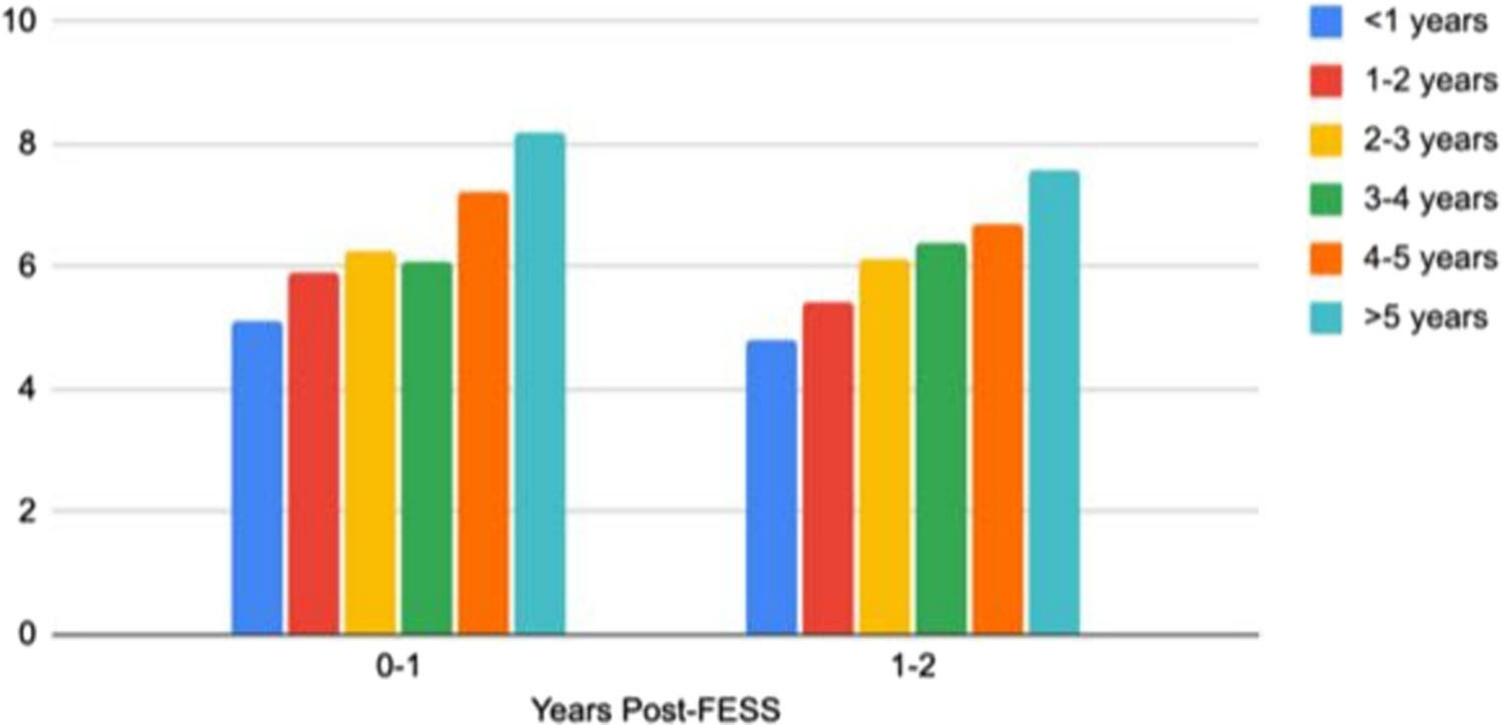
Mean number of sinusitis and nasal polyp‐related prescriptions.

We additionally estimated the healthcare utilization of each cohort within 2 years postoperatively. For the 2 years following FESS, we found that there was a significantly greater average number of instances of sinusitis/nasal polyps related prescriptions for all of the delayed FESS cohorts compared to the early FESS cohort (Figure 1).

## Discussion

In a previous study of a US‐based database, it was found that delayed FESS may negatively impact patient outcomes including a greater need for revision FESS and increased sinus‐related healthcare utilization.^9^ With the recent approval of T2 biologics for the treatment for CRSwNP,^15^, ^16^, ^17^ it is important to reassess the role of FESS timing on patient outcomes and need for additional sinus treatment. To our knowledge, this is the first study to reassess the relationship between FESS timing and disease management for patients with CRSwNP and to include prescription of T2 biologics in addition to revision FESS when assessing disease control.

Our study found that individuals undergoing delayed FESS (more than 5 years between initial diagnosis and FESS) had a similar odds of revision FESS compared to individuals undergoing FESS within the first year of CRSwNP diagnosis, with a statistically significant difference between groups only found between delayed (1‐2 years) and early FESS with the delayed (1‐2) FESS group having a 49% greater 5‐year odds of revision FESS. Although not statistically significant across other comparisons, this trend of increased need for revision FESS among individuals undergoing delayed FESS is consistent across all comparisons. This finding is consistent with previous literature suggesting a benefit to early FESS in reducing future need for intervention.^9^

Our findings demonstrate that individuals undergoing delayed (>5 years) FESS had a 96% increase in the 2‐year odds and 83% increase in the 5‐year odds of new T2 biologic prescription compared to individuals undergoing early FESS. This finding is in line with previous literature which suggests that early disease intervention reduces the need for future operative intervention. However, this is the first study to demonstrate the preservation of this trend in the context of T2 biologic prescription. We further assessed the combined odds of revision FESS and/or T2 biologic prescription, as both FESS and T2 biologics can be utilized for treatment of persistent CRSwNP symptoms not controlled with conservative management.^18^, ^19^ This allowed us to understand the overall control of disease achieved relative to the timing of FESS. Our analysis revealed that the timing of FESS was significantly associated with the need for further disease management within 5 years postsurgery, with a greater need for intervention observed in all delayed FESS groups. These findings suggest that while the timing of FESS may not influence the immediate management of CRSwNP, delayed FESS may require more extensive disease management in the long term, as evidenced by the need for additional CRSwNP‐targeted interventions.

Finally, we assessed the impact of FESS timing on the need for sinusitis and nasal‐polyp‐related prescriptions. Our findings are consistent with those found by Benninger et al., which similarly found a greater average number of CRSwNP related prescriptions in those who delayed FESS compared to those who underwent early FESS.^9^ This further suggests that a delay in FESS may result in an poorer overall prognosis, resulting in an increase in need for non‐surgical/biologic therapy for CRSwNP related disease.

While our study consistently demonstrates an increased need for CRSwNP‐related intervention associated with delays in primary FESS, we acknowledge that the shared patient‐physician decision to undergo or delay FESS is complex. In addition to patient and/or physician preferences, characteristics such as disease progression/severity and comorbidities such as those which may impact operative candidacy may contribute to inherent differences among patients undergoing early versus delayed FESS. While we are limited in our assessment of attitudes toward surgery and objective measures of disease severity (such as polyp grade) due to the nature of a retrospective, database study, we are able to further investigate patient comorbidities. Supplemental Tables S1‐5, available online demonstrate the differences in comorbidities found across each delayed FESS cohort when compared to the early FESS cohort. An SMD < 0.1 is typically indicative of balanced groups,^20^ and our assessment indicates that a majority of characteristics, such as diabetes, hypertension, obesity, sleep apnea, and ischemic heart disease are well balanced between the early and 1 to 2 year delayed cohorts. However, in comparison of the early cohort to the progressively delayed cohorts, the degree of comorbidity imbalance increases, with imbalance noted among nearly all assessed comorbidities. These findings suggest that patient comorbidities may be contributing to the decision to delay surgery. This finding is not surprising, given that increasing patient complexity may lead both the physician and patient to consider non‐surgical management. While we are unable to further adjust for the impact of these comorbidities on our outcome due to limitations of the TriNetX analysis platform, it is important to consider that the increased incidence of these comorbidities, including asthma in particular, among individuals undergoing delayed FESS may contribute to the increased odds of our measured outcomes. Future electronic medical record‐based studies capable of providing a granular analysis of factors driving delays in FESS and influencing biologic prescription are needed.

An important strength of this study includes its appropriate utilization of a large database to query disease management in a large sample of patients with CRSwNP. While this study design allows for a large‐scale analysis, similar to a previous database study of the impact of a delayed FESS on disease management,^9^ it also has important limitations. Although the TriNetX platform allows for longitudinal analysis of a patient′s treatment pathway, it is limited by utilization of ICD‐10 codes to identify patients and outcomes of interest. We are unable to assess other patient characteristics that may contribute to likelihood of undergoing further surgery, such as attitude toward surgical intervention. Furthermore, the prevalence of CRS patients with nasal polyps (ICD‐10 J33) was found to be 0.17% within the TriNetX platform, which is notably lower than the estimated prevalence of 1% to 4% in the US population.^21^ This may be reflective of the limitations of the TriNetX platform′s generalizability. Finally, it is important to note that this study includes data from years prior to when biologics were approved for the treatment of CRSwNP. Inclusion of this period of time likely results in our study underestimating the utilization of biologics after delayed FESS, as patients who may otherwise have been prescribed a biologic for further disease control were treated with alternate modalities such as revision FESS or adjunct medical therapy. Despite these limitations, our findings remain relevant to provide initial context to the relationship between FESS timing and CRSwNP disease management. Future prospective studies are needed to further investigate this association between FESS timing and CRSwNP‐specific disease control.

## Conclusions

This is the first study to assess the impact of surgical timing on the need for prescription of T2 biologics. Our findings suggest that delayed FESS is associated with a significantly greater odds of biologic prescription. Delayed FESS was also associated with a greater number of outpatient visits and prescriptions related to medical management. Further studies including sinonasal quality of life assessments are needed to better understand factors contributing to the observed relationship.

## Author Contributions

**Radhika Duggal**, data analysis, manuscript writing and reviewing; **Trisha Shang**, data acquisition and analysis, manuscript writing and reviewing; **David R. Hoying**, data acquisition and analysis, manuscript writing and reviewing; **David Kaelber**, data acquisition, manuscript reviewing; **Mohamad R. Chaaban**, conceptualization, data analysis, manuscript writing and reviewing.

## Disclosures

### Competing interests

None.

### Funding sources

None.

## Supporting information

supporting information.the supplemental materials outline the comparative demographics before and after propensity score matching as well as the specific cpt codes utilized to define the cohorts and outcomes in this study.
