# Supplementary material for: Impact of Early Versus Delayed Functional Endoscopic Sinus Surgery for Chronic Rhinosinusitis With Nasal Polyps
Source: Otolaryngol Head Neck Surg. 2026 Jan 19;174(3):671–7. doi: 10.1002/ohn.70131 (PMC12948396; doi:10.1002/ohn.70131)
Supplement: Supplementary file 1 — supporting information.the supplemental materials outline the comparative demographics before and after propensity score matching as well as the specific cpt codes utilized to define the cohorts and outcomes in this study. [file OHN-174-671-s001.docx]

**Supplemental Materials**

**Tables**

**Table 1: Propensity Score Matching Results Between CRSwNP 1-2 years versus < 1 year.**

| **Characteristics** | **Before Matching** | | | **After Matching** | | |
| --- | --- | --- | --- | --- | --- | --- |
|  | **CRSwNP 1-2 yrs** | **CRSwNP < 1 yr** | **SMD** | **CRSwNP 1-2 yrs** | **CRSwNP < 1 yr** | **SMD** |
| Age, mean (SD), y | 48 ± 17 | 47 ± 17.5 | 0.06 | 48 ± 17 | 48 ± 17 | <0.001 |
| **Sex** | | | | | | |
| Male | 58.1% | 59.8% | 0.035 | 58.1% | 58.1% | <0.001 |
| **Comorbidities** | | | | | | |
| Asthma | 45.3% | 33.7% | 0.24 | 45.3% | 33.3% | 0.25 |
| Depressive episode | 12% | 9.9% | 0.07 | 12% | 10.6% | 0.05 |
| Diabetes Mellitus | 11.9% | 10.7% | 0.04 | 11.9% | 10.8% | 0.04 |
| Allergic rhinitis, unspecified | 39.6% | 27.9% | 0.25 | 39.6% | 26.8% | 0.27 |
| Chronic ischemic heart disease | 7.2% | 6.1% | 0.04 | 7.2% | 5.8% | 0.06 |
| Primary hypertension | 32.1% | 29% | 0.07 | 32.1% | 30% | 0.05 |
| Type 2 Diabetes Mellitus | 11.3% | 10.4% | 0.03 | 11.3% | 10.5% | 0.03 |
| Sleep apnea | 15.9% | 14.1% | 0.05 | 15.9% | 12.6% | 0.09 |
| Disease of circulatory system | 42.6% | 38.2% | 0.09 | 42.6% | 40.4% | 0.05 |
| Normal weight (BMI<30) | 31.5% | 31.2% | 0.01 | 31.5% | 30.5% | 0.02 |
| **Procedures** | | | | | | |
| Maxillary Antrostomy w/ tissue removal (31267) | 64.4% | 68.6% | 0.09 | 64.4% | 69.5% | 0.11 |
| Frontal Sinus w/ tissue removal (31276) | 52.5% | 51.3% | 0.02 | 52.5% | 50.5% | 0.04 |
| Total Ethmoidectomy (31255) | 39% | 39.2% | 0.004 | 39% | 41% | 0.04 |
| Maxillary Antrostomy (31256) | 31.2% | 28% | 0.07 | 31.2% | 28% | 0.07 |
| Total Ethmoidectomy and Sphenoidotomy w/ removal of tissue (31259) | 27.3% | 25.7% | 0.04 | 27.3% | 25.1% | 0.05 |
| Sphenoidotomy w/ removal of tissue (31288) | 13.7% | 16.5% | 0.08 | 13.7% | 18% | 0.12 |
| Sphenoidotomy (31287) | 17.1% | 15.7% | 0.04 | 17% | 16% | 0.04 |
| Total ethmoidectomy with frontal sinus exploration w/ removal of tissue (31253) | 15.2% | 15.3% | 0.004 | 15.2% | 15.3% | 0.002 |
| Partial Ethmoidectomy (31254) | 11.3% | 13.9% | 0.08 | 11.3% | 14.5% | 0.09 |
| Total Ethmoidectomy and Sphenoidotomy (31257) | 11.8% | 11.2% | 0.02 | 11.8% | 10.2% | 0.05 |

**Table 2: Propensity Score Matching Results Between CRSwNP 2-3 years versus < 1 year.**

| **Characteristics** | **Before Matching** | | | **After Matching** | | |
| --- | --- | --- | --- | --- | --- | --- |
|  | **CRSwNP 2-3 yrs** | **CRSwNP < 1 yr** | **SMD** | **CRSwNP 2-3 yrs** | **CRSwNP < 1 yr** | **SMD** |
| Age, mean (SD), y | 48.5 ± 16.9 | 47 ± 17.5 | 0.09 | 48.5 ± 16.9 | 48.5 ± 16.9 | <0.001 |
| **Sex** | | | | | | |
| Male | 55.9% | 59.8% | 0.08 | 55.9% | 55.9% | <0.001 |
| **Comorbidities** | | | | | | |
| Asthma | 48.4% | 33.7% | 0.30 | 48.4% | 33.6% | 0.31 |
| Depressive episode | 14.5% | 9.9% | 0.14 | 14.5% | 11.3% | 0.10 |
| Diabetes Mellitus | 14.3% | 10.7% | 0.11 | 14.3% | 9.8% | 0.14 |
| Allergic rhinitis, unspecified | 45.3% | 27.9% | 0.37 | 45.3% | 28.4% | 0.36 |
| Chronic ischemic heart disease | 8.4% | 6.1% | 0.09 | 8.4% | 5.7% | 0.10 |
| Primary hypertension | 35.5% | 29% | 0.14 | 35.5% | 31.4% | 0.90 |
| Type 2 Diabetes Mellitus | 13.6% | 10.4% | 0.10 | 13.6% | 9.6% | 0.13 |
| Sleep apnea | 18.3% | 14.1% | 0.11 | 18.3% | 12.7% | 0.15 |
| Disease of circulatory system | 46% | 38.2% | 0.16 | 46% | 43.4% | 0.05 |
| Normal weight (BMI <30) | 33.3% | 31.2% | 0.05 | 33.3% | 29.8% | 0.08 |
| **Procedures** | | | | | | |
| Maxillary Antrostomy w/ tissue removal (31267) | 64.8% | 68.6% | 0.08 | 64.8% | 68.8% | 0.09 |
| Frontal Sinus w/ tissue removal (31276) | 52.3% | 51.3% | 0.02 | 52.3% | 49.8% | 0.05 |
| Total Ethmoidectomy (31255) | 39.9% | 39.2% | 0.01 | 39.9% | 39.2% | 0.01 |
| Maxillary Antrostomy (31256) | 27.4% | 28% | 0.01 | 27.4% | 28.4% | 0.02 |
| Total Ethmoidectomy and Sphenoidotomy w/ removal of tissue (31259) | 27% | 25.7% | 0.03 | 27% | 25.4% | 0.04 |
| Sphenoidotomy w/ removal of tissue (31288) | 17.4% | 16.5% | 0.02 | 17.4% | 17.2% | 0.01 |
| Sphenoidotomy (31287) | 15.3% | 15.7% | 0.01 | 15.3% | 16.4% | 0.03 |
| Total ethmoidectomy with frontal sinus exploration w/ removal of tissue (31253) | 14.1% | 15.3% | 0.03 | 14.1% | 13.6% | 0.02 |
| Partial Ethmoidectomy (31254) | 11.8% | 13.9% | 0.06 | 11.8% | 15.9% | 0.12 |
| Total Ethmoidectomy and Sphenoidotomy (31257) | 14.5% | 11.2% | 0.10 | 14.5% | 10.8% | 0.11 |

**Table 3: Propensity Score Matching Results Between CRSwNP 3-4 years versus < 1 year.**

| **Characteristics** | **Before Matching** | | | **After Matching** | | |
| --- | --- | --- | --- | --- | --- | --- |
|  | **CRSwNP 3-4 yrs** | **CRSwNP < 1 yr** | **SMD** | **CRSwNP 3-4 yrs** | **CRSwNP < 1 yr** | **SMD** |
| Age, mean (SD), y | 49.4 ± 17.1 | 47 ± 17.5 | 0.14 | 49.4 ± 17.1 | 49.4 ± 17.1 | <0.001 |
| **Sex** | | | | | | |
| Male | 59.5% | 59.8% | 0.01 | 59.5% | 59.5% | <0.001 |
| **Comorbidities** | | | | | | |
| Asthma | 52% | 33.7% | 0.38 | 52% | 31.5% | 0.43 |
| Depressive episode | 14.4% | 9.9% | 0.14 | 14.4% | 12% | 0.07 |
| Diabetes Mellitus | 13.7% | 10.7% | 0.09 | 13.7% | 10.7% | 0.09 |
| Allergic rhinitis, unspecified | 46.1% | 27.9% | 0.38 | 46.1% | 29.3% | 0.35 |
| Chronic ischemic heart disease | 6.6% | 6.1% | 0.02 | 6.6% | 5.6% | 0.04 |
| Primary hypertension | 38.3% | 29% | 0.20 | 38.3% | 32.4% | 0.12 |
| Type 2 Diabetes Mellitus | 13.4% | 10.4% | 0.09 | 13.4% | 10.7% | 0.08 |
| Sleep apnea | 18.3% | 14.1% | 0.11 | 18.3% | 13.4% | 0.13 |
| Disease of circulatory system | 46.8% | 38.2% | 0.18 | 46.8% | 44.4% | 0.05 |
| Normal weight (BMI <30) | 37.6% | 31.2% | 0.14 | 37.6% | 30.5% | 0.15 |
| **Procedures** | | | | | | |
| Maxillary Antrostomy w/ tissue removal (31267) | 62.2% | 68.6% | 0.13 | 62.2% | 72% | 0.21 |
| Frontal Sinus w/ tissue removal (31276) | 49.5% | 51.3% | 0.04 | 49.5% | 51% | 0.03 |
| Total Ethmoidectomy (31255) | 43.2% | 39.2% | 0.80 | 43.2% | 42.9% | 0.005 |
| Maxillary Antrostomy (31256) | 26.8% | 28% | 0.03 | 26.8% | 28.3% | 0.33 |
| Total Ethmoidectomy and Sphenoidotomy w/ removal of tissue (31259) | 24.4% | 25.7% | 0.08 | 24.4% | 25.9% | 0.03 |
| Sphenoidotomy w/ removal of tissue (31288) | 16.1% | 16.5% | 0.01 | 16.1% | 17.6% | 0.04 |
| Sphenoidotomy (31287) | 18% | 15.7% | 0.06 | 18% | 16.6% | 0.04 |
| Total ethmoidectomy with frontal sinus exploration w/ removal of tissue (31253) | 16.8% | 15.3% | 0.04 | 16.8% | 14.1% | 0.07 |
| Partial Ethmoidectomy (31254) | 9.3% | 13.9% | 0.15 | 9.3% | 14.9% | 0.17 |
| Total Ethmoidectomy and Sphenoidotomy (31257) | 12% | 11.2% | 0.023 | 12% | 10.7% | 0.40 |

**Table 4: Propensity Score Matching Results Between CRSwNP 4-5 years versus < 1 year.**

| **Characteristics** | **Before Matching** | | | **After Matching** | | |
| --- | --- | --- | --- | --- | --- | --- |
|  | **CRSwNP 4-5 yrs** | **CRSwNP < 1 yr** | **SMD** | **CRSwNP 4-5 yrs** | **CRSwNP < 1 yr** | **SMD** |
| Age, mean (SD), y | 51± 16.6 | 47 ± 17.5 | 0.24 | 51± 16.6 | 51± 16.6 | <0.001 |
| **Sex** | | | | | | |
| Male | 57.6% | 59.8% | 0.04 | 57.6% | 57.6% | <0.001 |
| **Comorbidities** | | | | | | |
| Asthma | 52.7% | 33.7% | 0.40 | 52.7% | 30.4% | 0.46 |
| Depressive episode | 16.3% | 9.9% | 0.19 | 16.3% | 9.5% | 0.20 |
| Diabetes Mellitus | 14.8% | 10.7% | 0.12 | 14.8% | 12.4% | 0.07 |
| Allergic rhinitis, unspecified | 48.8% | 27.9% | 0.44 | 48.8% | 28.3% | 0.43 |
| Chronic ischemic heart disease | 9.5% | 6.1% | 0.13 | 9.5% | 8.1% | 0.05 |
| Primary hypertension | 38.9% | 29% | 0.21 | 38.9% | 32.5% | 0.13 |
| Type 2 Diabetes Mellitus | 14.1% | 10.4% | 0.11 | 14.1% | 12.4% | 0.05 |
| Sleep apnea | 19.4% | 14.1% | 0.14 | 19.4% | 13.4% | 0.16 |
| Disease of circulatory system | 46.6% | 38.2% | 0.17 | 46.6% | 47% | 0.01 |
| Normal weight (BMI <30) | 41.7% | 31.2% | 0.22 | 41.7% | 29.3% | 0.26 |
| **Procedures** | | | | | | |
| Maxillary Antrostomy w/ tissue removal (31267) | 59.7% | 68.6% | 0.19 | 59.7% | 74.2% | 0.31 |
| Frontal Sinus w/ tissue removal (31276) | 53% | 51.3% | 0.03 | 53% | 47.7% | 0.11 |
| Total Ethmoidectomy (31255) | 47% | 39.2% | 0.16 | 47% | 38.9% | 0.17 |
| Maxillary Antrostomy (31256) | 30.4% | 28% | 0.05 | 30.4% | 25% | 0.12 |
| Total Ethmoidectomy and Sphenoidotomy w/ removal of tissue (31259) | 20.8% | 25.7% | 0.12 | 20.8% | 25.8% | 0.12 |
| Sphenoidotomy w/ removal of tissue (31288) | 15.2% | 16.5% | 0.04 | 15.2% | 18% | 0.08 |
| Sphenoidotomy (31287) | 21.2% | 15.7% | 0.14 | 21.2% | 17.7% | 0.09 |
| Total ethmoidectomy with frontal sinus exploration w/ removal of tissue (31253) | 14.8% | 15.3% | 0.01 | 14.8% | 14.8% | <0.001 |
| Partial Ethmoidectomy (31254) | 12.4% | 13.9% | 0.01 | 12.4% | 17.3% | 0.14 |
| Total Ethmoidectomy and Sphenoidotomy (31257) | 11.7% | 11.2% | 0.14 | 11.7% | 11% | 0.02 |

**Table 5: Propensity Score Matching Results Between CRSwNP > 5 years versus < 1 year.**

| **Characteristics** | **Before Matching** | | | **After Matching** | | |
| --- | --- | --- | --- | --- | --- | --- |
|  | **CRSwNP >5 yrs** | **CRSwNP < 1 yr** | **SMD** | **CRSwNP >5 yrs** | **CRSwNP < 1 yr** | **SMD** |
| Age, mean (SD), y | 53.1 ± 15.6 | 47 ± 17.5 | 0.37 | 53.1 ± 15.6 | 53.1 ± 15.6 | <0.001 |
| **Sex** | | | | | | |
| Male | 56.2% | 59.8% | 0.07 | 56.2% | 56.2% | <0.001 |
| **Comorbidities** | | | | | | |
| Asthma | 60.2% | 33.7% | 0.55 | 60.2% | 34% | 0.54 |
| Depressive episode | 21.9% | 9.9% | 0.33 | 21.9% | 10.9% | 0.30 |
| Diabetes Mellitus | 17.5% | 10.7% | 0.19 | 17.5% | 13% | 0.12 |
| Allergic rhinitis, unspecified | 57.6% | 27.9% | 0.62 | 57.6% | 26.4% | 0.70 |
| Chronic ischemic heart disease | 10.8% | 6.1% | 0.17 | 10.8% | 7% | 0.13 |
| Primary hypertension | 41.8% | 29% | 0.27 | 41.8% | 35% | 0.14 |
| Type 2 Diabetes Mellitus | 16.1% | 10.4% | 0.17 | 16.1% | 12.7% | 0.10 |
| Sleep apnea | 23.3% | 14.1% | 0.24 | 23.3% | 15.4% | 0.20 |
| Disease of circulatory system | 60.2% | 38.2% | 0.45 | 60.2% | 45.8% | 0.29 |
| Normal weight (BMI <30) | 44.3% | 31.2% | 0.27 | 44.3% | 32.3% | 0.25 |
| **Procedures** | | | | | | |
| Maxillary Antrostomy w/ tissue removal (31267) | 64.9% | 68.6% | 0.08 | 64.9% | 69.1% | 0.09 |
| Frontal Sinus w/ tissue removal (31276) | 50.2% | 51.3% | 0.02 | 50.2% | 52.7% | 0.05 |
| Total Ethmoidectomy (31255) | 32% | 39.2% | 0.15 | 32% | 40.5% | 0.18 |
| Maxillary Antrostomy (31256) | 30% | 28% | 0.05 | 30% | 28% | 0.05 |
| Total Ethmoidectomy and Sphenoidotomy w/ removal of tissue (31259) | 27% | 25.7% | 0.03 | 27% | 25.7% | 0.03 |
| Sphenoidotomy w/ removal of tissue (31288) | 13.7% | 16.5% | 0.08 | 13.7% | 17.5% | 0.11 |
| Sphenoidotomy (31287) | 15.6% | 15.7% | 0.002 | 15.6% | 16.3% | 0.02 |
| Total ethmoidectomy with frontal sinus exploration w/ removal of tissue (31253) | 18.1% | 15.3% | 0.08 | 18.1% | 14.2% | 0.11 |
| Partial Ethmoidectomy (31254) | 11.1% | 13.9% | 0.08 | 11.1% | 14% | 0.09 |
| Total Ethmoidectomy and Sphenoidotomy (31257) | 14.3% | 11.2% | 0.09 | 14.3% | 10.4% | 0.12 |

**Patient Cohorts**

1. **Persons in < 1-year cohort were defined as any person with a first instance ICD-10 Diagnosis of CRS (J32) with the following first-instance Logical Observation Identifiers, Names, and Codes (LOINC) within 1 year:**
   1. Sphenoidotomy; with the removal of tissue from the sphenoid sinus (CPT: 31288) OR
   2. Sphenoidotomy (CPT: 31287) OR
   3. Ethmoidectomy; total (CPT: 31255) OR
   4. Maxillary Antrostomy (CPT: 31256) OR
   5. Ethmoidectomy; partial (CPT: 31254) OR
   6. Ethmoidectomy; total, including frontal sinus exploration, with the removal of tissue from the frontal sinus, when performed (CPT: 31253) OR
   7. Ethmoidectomy; total, including sphenoidotomy (CPT: 31257) OR
   8. Nasal/sinus endoscopy, surgical with ethmoidectomy; total (anterior and posterior), including sphenoidotomy, with removal of tissue from the sphenoid sinus (CPT: 31259) OR
   9. Maxillary antrostomy, with the removal of tissue from the maxillary sinus (CPT: 31267) OR
   10. Frontal sinus exploration, including removal of tissue from the frontal sinus, when performed (CPT: 31276).
2. **Persons in the 1-2 years cohort were defined as any person with a first instance ICD-10 Diagnosis of CRS (J32) with the following first-instance Logical Observation Identifiers, Names, and Codes (LOINC) 1-2 years after the CRS diagnosis:**
   1. Sphenoidotomy; with the removal of tissue from the sphenoid sinus (CPT: 31288) OR
   2. Sphenoidotomy (CPT: 31287) OR
   3. Ethmoidectomy; total (CPT: 31255) OR
   4. Maxillary Antrostomy (CPT: 31256) OR
   5. Ethmoidectomy; partial (CPT: 31254) OR
   6. Ethmoidectomy; total, including frontal sinus exploration, with the removal of tissue from the frontal sinus, when performed (CPT: 31253) OR
   7. Ethmoidectomy; total, including sphenoidotomy (CPT: 31257) OR
   8. Nasal/sinus endoscopy, surgical with ethmoidectomy; total (anterior and posterior), including sphenoidotomy, with removal of tissue from the sphenoid sinus (CPT: 31259) OR
   9. Maxillary antrostomy, with the removal of tissue from the maxillary sinus (CPT: 31267) OR
   10. Frontal sinus exploration, including removal of tissue from the frontal sinus, when performed (CPT: 31276).
3. **Persons in the 2-3 years cohort were defined as any person with a first instance ICD-10 Diagnosis of CRS (J32) with the following first-instance Logical Observation Identifiers, Names, and Codes (LOINC) 2-3 years after the CRS diagnosis:**
   1. Sphenoidotomy; with the removal of tissue from the sphenoid sinus (CPT: 31288) OR
   2. Sphenoidotomy (CPT: 31287) OR
   3. Ethmoidectomy; total (CPT: 31255) OR
   4. Maxillary Antrostomy (CPT: 31256) OR
   5. Ethmoidectomy; partial (CPT: 31254) OR
   6. Ethmoidectomy; total, including frontal sinus exploration, with the removal of tissue from the frontal sinus, when performed (CPT: 31253) OR
   7. Ethmoidectomy; total, including sphenoidotomy (CPT: 31257) OR
   8. Nasal/sinus endoscopy, surgical with ethmoidectomy; total (anterior and posterior), including sphenoidotomy, with removal of tissue from the sphenoid sinus (CPT: 31259) OR
   9. Maxillary antrostomy, with the removal of tissue from the maxillary sinus (CPT: 31267) OR
   10. Frontal sinus exploration, including removal of tissue from the frontal sinus, when performed (CPT: 31276).
4. **Persons in the 3-4 years cohort were defined as any person with a first instance ICD-10 Diagnosis of CRS (J32) with the following first-instance Logical Observation Identifiers, Names, and Codes (LOINC) 3-4 years after the CRS diagnosis:**
   1. Sphenoidotomy; with the removal of tissue from the sphenoid sinus (CPT: 31288) OR
   2. Sphenoidotomy (CPT: 31287) OR
   3. Ethmoidectomy; total (CPT: 31255) OR
   4. Maxillary Antrostomy (CPT: 31256) OR
   5. Ethmoidectomy; partial (CPT: 31254) OR
   6. Ethmoidectomy; total, including frontal sinus exploration, with the removal of tissue from the frontal sinus, when performed (CPT: 31253) OR
   7. Ethmoidectomy; total, including sphenoidotomy (CPT: 31257) OR
   8. Nasal/sinus endoscopy, surgical with ethmoidectomy; total (anterior and posterior), including sphenoidotomy, with removal of tissue from the sphenoid sinus (CPT: 31259) OR
   9. Maxillary antrostomy, with the removal of tissue from the maxillary sinus (CPT: 31267) OR
   10. Frontal sinus exploration, including removal of tissue from the frontal sinus, when performed (CPT: 31276).
5. **Persons in the 4-5 years cohort were defined as any person with a first instance ICD-10 Diagnosis of CRS (J32) with the following first-instance Logical Observation Identifiers, Names, and Codes (LOINC) 4-5 years after the CRS diagnosis:**
   1. Sphenoidotomy; with the removal of tissue from the sphenoid sinus (CPT: 31288) OR
   2. Sphenoidotomy (CPT: 31287) OR
   3. Ethmoidectomy; total (CPT: 31255) OR
   4. Maxillary Antrostomy (CPT: 31256) OR
   5. Ethmoidectomy; partial (CPT: 31254) OR
   6. Ethmoidectomy; total, including frontal sinus exploration, with the removal of tissue from the frontal sinus, when performed (CPT: 31253) OR
   7. Ethmoidectomy; total, including sphenoidotomy (CPT: 31257) OR
   8. Nasal/sinus endoscopy, surgical with ethmoidectomy; total (anterior and posterior), including sphenoidotomy, with removal of tissue from the sphenoid sinus (CPT: 31259) OR
   9. Maxillary antrostomy, with the removal of tissue from the maxillary sinus (CPT: 31267) OR
   10. Frontal sinus exploration, including removal of tissue from the frontal sinus, when performed (CPT: 31276).
6. **Persons in the > 5 years cohort were defined as any person with a first instance ICD-10 Diagnosis of CRS (J32) with the following first-instance Logical Observation Identifiers, Names, and Codes (LOINC) > 5 years after the CRS diagnosis:**
   1. Sphenoidotomy; with the removal of tissue from the sphenoid sinus (CPT: 31288) OR
   2. Sphenoidotomy (CPT: 31287) OR
   3. Ethmoidectomy; total (CPT: 31255) OR
   4. Maxillary Antrostomy (CPT: 31256) OR
   5. Ethmoidectomy; partial (CPT: 31254) OR
   6. Ethmoidectomy; total, including frontal sinus exploration, with the removal of tissue from the frontal sinus, when performed (CPT: 31253) OR
   7. Ethmoidectomy; total, including sphenoidotomy (CPT: 31257) OR
   8. Nasal/sinus endoscopy, surgical with ethmoidectomy; total (anterior and posterior), including sphenoidotomy, with removal of tissue from the sphenoid sinus (CPT: 31259) OR
   9. Maxillary antrostomy, with the removal of tissue from the maxillary sinus (CPT: 31267) OR
   10. Frontal sinus exploration, including removal of tissue from the frontal sinus, when performed (CPT: 31276).

**Patient Demographics**

**The following codes were used for patient demographic comparisons between cohorts.**

1. Asthma (ICD10CM: J45)
2. Depressive episode (ICD10CM: F32)
3. Diabetes mellitus (ICD10CM: E08-E13)
4. Allergic rhinitis, unspecified (ICD10CM: J30.9)
5. Chronic ischemic heart disease (ICD10CM: I25)
6. Essential primary hypertension (ICD10CM: I10)
7. Type 2 diabetes mellitus (ICD10CM: E11)
8. Sleep apnea (ICD10CM: G47.3)
9. Diseases of the circulatory system (ICD10CM: I00-I99)
10. Normal Weight (BMI <30) LOINC: 39156-5)

**Patient Outcomes**

1. **Revision Surgery Rates**

**The number of the following Logical Observation Identifiers, Names, and Codes (LOINC) within 2 years or 5 years of initial surgery:**

1. Sphenoidotomy; with the removal of tissue from the sphenoid sinus (CPT: 31288) OR
2. Sphenoidotomy (CPT: 31287) OR
3. Ethmoidectomy; total (CPT: 31255) OR
4. Maxillary Antrostomy (CPT: 31256) OR
5. Ethmoidectomy; partial (CPT: 31254) OR
6. Ethmoidectomy; total, including frontal sinus exploration, with the removal of tissue from the frontal sinus, when performed (CPT: 31253) OR
7. Ethmoidectomy; total, including sphenoidotomy (CPT: 31257) OR
8. Nasal/sinus endoscopy, surgical with ethmoidectomy; total (anterior and posterior), including sphenoidotomy, with removal of tissue from the sphenoid sinus (CPT: 31259) OR
9. Maxillary antrostomy, with the removal of tissue from the maxillary sinus (CPT: 31267) OR
10. Frontal sinus exploration, including removal of tissue from the frontal sinus, when performed (CPT: 31276).
11. **First-time Biologic prescription**

**The number of the following RxNorm Codes within 2 years or 5 years of initial surgery (excluding codes prior to the initial surgery):**

1. Dupilumab (RxNorm: 1876376) OR
2. Mepolizumab (RxNorm: 1720597) OR
3. Omalizumab (RxNorm: 302379)

**3. Combined rates of revision surgery and biologic prescriptions**

**The number of the following CPT or RxNorm codes within 2 years or 5 years of initial surgery:**

1. Sphenoidotomy; with the removal of tissue from the sphenoid sinus (CPT: 31288) OR
2. Sphenoidotomy (CPT: 31287) OR
3. Ethmoidectomy; total (CPT: 31255) OR
4. Maxillary Antrostomy (CPT: 31256) OR
5. Ethmoidectomy; partial (CPT: 31254) OR
6. Ethmoidectomy; total, including frontal sinus exploration, with the removal of tissue from the frontal sinus, when performed (CPT: 31253) OR
7. Ethmoidectomy; total, including sphenoidotomy (CPT: 31257) OR
8. Nasal/sinus endoscopy, surgical with ethmoidectomy; total (anterior and posterior), including sphenoidotomy, with removal of tissue from the sphenoid sinus (CPT: 31259) OR
9. Maxillary antrostomy, with the removal of tissue from the maxillary sinus (CPT: 31267) OR
10. Frontal sinus exploration, including removal of tissue from the frontal sinus, when performed (CPT: 31276).
11. Dupilumab (RxNorm: 1876376) OR
12. Mepolizumab (RxNorm: 1720597) OR
13. Omalizumab (RxNorm: 302379)

**4. Drug therapeutic class prescriptions**

**The number of the following VA, ATC, and RxNorm codes between 0-1 year and 1-2 years of initial surgery:**

- 1. Antihistamines (VA: AH000)
  2. Antitussives/Expectorants (VA: RE300)
  3. Antihistamine/antitussive (VA: RE507)
  4. Antimicrobials (VA: AM000)
  5. Antiasthma/bronchodilators (VA: RE100)
  6. Corticosteroids (ATC: R01AD)
  7. Leukotriene receptor antagonists (ATC: R03DC)
  8. Analgesics (VA: CN100)
  9. Fluticasone (RxNorm: 41126)
  10. Beclomethasone (RxNorm: 1347)
  11. Triamcinolone (RxNorm: 10759)
  12. Budesonide (RxNorm: 19831)
  13. Mometasone (RxNorm: 108118)
  14. Ciclesonide (RxNorm: 274964)
